# Effects of qigong on systolic and diastolic blood pressure lowering: a systematic review with meta-analysis and trial sequential analysis

**DOI:** 10.1186/s12906-020-03172-3

**Published:** 2021-01-06

**Authors:** Siew Mooi Ching, Naidu Ragubathi Mokshashri, Maharajan Mari Kannan, Kai Wei Lee, Nurin Amalina Sallahuddin, Jun Xun Ng, Jie Lin Wong, Navin Kumar Devaraj, Fan Kee Hoo, Yee Shen Loo, Sajesh K. Veettil

**Affiliations:** 1 Department of Family Medicine, Faculty of Medicine and Health Sciences, Universiti Putra Malaysia, 43400 Serdang, Selangor Malaysia; 2 Malaysian Research Institute on Ageing, Universiti Putra Malaysia, 43400 Serdang, Selangor Malaysia; 3 School of Pharmacy/School of Postgraduate Studies, International Medical University, Kuala Lumpur, 57000 Malaysia; 4 Department of Pre-Clinical Sciences, Faculty of Medicine and Health Sciences, Universiti Tunku Abdul Rahman, 43000 Kajang, Selangor Malaysia; 5 Department of Neurology, Faculty of Medicine and Health Sciences, Universiti Putra Malaysia, 43400 Serdang, Selangor Malaysia; 6 Department of Pharmacotherapy, College of Pharmacy, University of Utah, 30 2000 E, Salt Lake City, UT 84112 USA

**Keywords:** Hypertension, Blood pressure, Qigong, Complementary therapies, Systematic review, meta-analysis, randomized controlled trials, trial sequential analysis

## Abstract

**Background:**

The benefits of qigong for systolic and diastolic blood pressure (BP) reduction have been noted in previously published systematic reviews; however, the data on its effectiveness has been at best scarce. We aimed to update the evidence of qigong on blood pressure reduction after taking into consideration the risks of random error and reliability of data in the cumulative meta-analysis using trial sequential analysis (TSA).

**Methods:**

Included trials were assessed using Cochrane risk of bias instrument. We performed meta-analysis with random-effects model and random errors were evaluated with TSA. We performed the search for the eligible randomized controlled trial (RCT) through Medline, Cinahl, Cochrane Central Register of Controlled Trials and also PubMed.

**Results:**

A total of 370 subjects sourced from seven eligible RCTs were entered into the analysis. The pooled results demonstrated the significant reduction with the use of qigong of the systolic blood pressure [weighted mean difference (WMD), − 10.66 mmHg (95% confidence interval (CI) = − 17.69,-3.62, *p* < 0.001] and diastolic BP [WMD, − 6.76 mmHg, 95% CI = − 12.22, − 1.30, *p* < 0.001] as compared to the control group.

**Conclusions:**

Significant reductions in BP is seen with the use of qigong as compared with the control group, suggesting that qigong may be used as a complementary therapy in the somewhat complicated management of hypertension.

**Supplementary Information:**

The online version contains supplementary material available at 10.1186/s12906-020-03172-3.

## Background

Cardiovascular disease accounts for one third of deaths worldwide and remains a growing public health problem [1]. Hypertension, a serious medical condition is a major contributor to cardiovascular diseases [2]. Considered as a ‘silent killer’, hypertension is firmly established in developed countries and the escalation in number of hypertension cases is advancing rapidly in developing countries [3]. The World Health Organization estimated that among the 1.13 billion people who have hypertension, less than 1 in 5 have it under good control [4]. Controlling blood pressure in patients diagnosed with hypertension is crucial to reduce cardiovascular events and mortality, especially among the middle-aged and older non-diabetic adults who are at high risk for cardiovascular diseases [5]. Maintaining blood pressure at recommended levels and aggressive management of hypertension can help prevent complications and even lower the risk of a cardiac event or stroke [6–9]. A few modifiable risk factors, such as unhealthy diets, obesity, physical inactivity and the consumption of alcohol and tobacco also play an adverse role in the progression of hypertension and related cardiovascular diseases [10, 11].

There are a variety of treatment options that are available to manage hypertension, which include the various classes of anti-hypertensive medications. The huge number of anti-hypertensive medication coupled with patient-specific requirements to treat hypertension, require more evidence-based approaches in order to recommend a more personalized therapeutic regimen [12]. Despite the availability of numerous pharmacological agents, blood pressure remains uncontrolled in two-thirds of patients who are only receiving pharmacological treatment for their hypertension [13]. Furthermore, the underlying cause remains unclear for the majority of hypertension cases. Studies have confirmed that long term stress or emotional distress can lead to blood vessel constriction, faster heart beats and reduction in blood flow to the peripheral body which then may predispose to the development and progression of hypertension [14]. Thus, there has been a revival of interest in using the available complementary and alternative medicine (CAM) approaches in the management of hypertension [15, 16].

Qigong, a combination of aerobic, isometric, and isotonic elements that is tagged along with meditation and relaxation exercises, have been shown to lead to improvements in physical health [17]. It is a common ancient healing technique originating in Oriental countries and has been used clinically for preventing and curing diseases, as well as for improving and maintaining overall health in these countries [18]. Ancient times have described qigong to be an effective traditional therapy and has been widely incorporated to medical treatment and health preservation since then. Traditionally, qigong has been defined as the harmony of *qi* (the internal vital energy of the body) and blood in the body, thus achieving the purpose of preventing disease, improving health and accelerating the recovery of limb function. It is a kind of body exercise that involves breathing, massaging through stretching and twisting arms and legs, and mental focus which act together to direct the flow of *qi*. It is also characterized as “Meditative Movement”, which is defined by Larkey et al. as a practice involving movement, a meditative state of mind, attention to the breath and deep relaxation [19]. It is also alternatively proposed as a movement-based embodied contemplative practice with a mechanism of becoming reflectively attentive to bodily sensations and sensory experiences [20]. Some even define it as mindfulness-based meditation practice known to engage selective brain areas and neural networks involved in attention, body awareness, emotion regulation and the sense of self [21].

Qigong is a low joint-impact exercise that is easy to practise and needs no special equipment and has been useful in patients with essential hypertension when compared to non-treated controls [22]. A previous study involving middle-aged individuals with hypertension provided evidence on the effect of qigong, which produced a significant reduction in resting blood pressure over a 10-week period with the frequency of three times a week training [23]. Qigong training at a frequency of 5-times per week for a total period of 12-weeks elicited even more reductions in resting blood pressure and improved the quality of life in the elderly population [24]. Furthermore, qigong has also been shown to effectively reduce blood pressure and at the same time, catecholamine levels in patients with essential hypertension [25]. One of the possible reasons for this positive effect is that it has the ability to increase blood flow throughout the body by the application of repetitive movements that relieve pathological stagnation and up-regulate the function of the visceral organs [26]. The effectiveness of qigong on blood pressure (BP) control have been previously reported in systematic reviews [26, 27]. Despite the collective positive evidence supporting the effectiveness of qigong practice in lowering the blood pressure and improving the quality of life, their relative effectiveness is not well established.

Published systematic reviews and meta-analyses based on the analysis of randomised controlled trials (RCTs) have proposed that qigong is effective in helping better manage hypertension. When a meta-analysis comprises a small number of RCTs and patients, random errors can often lead to a deceptive conclusion [28]. This emphasizes the importance of updating the summary of effects of qigong on hypertension using recently published trials and taking into account the risks of random errors. While understanding the benefits of qigong is important in the development of preventive measures and supporting its role as a complementary therapy to the current treatment strategies of hypertension, equally important is recognizing its effectiveness as a tool to promote prevention of cardiovascular complications. Hence, we conducted this study to evaluate the available evidence on the effects of qigong on hypertension in order to summarize and synthesize the clinical evidence available from RCTs on the comparative effectiveness of qigong exercises on hypertension using the combined systematic review and trial sequential analysis methodology.

## Methods

### Protocol approval and data sources

The protocol was registered with the Medical Research and Ethics Committee, Ministry of Health Malaysia (NMRR-20-330-53,097). In conducting this meta-analysis, the Cochrane Handbook for Systematic Reviews of Interventions was used as a guide [23] to ensure the compliance of this study with the PRISMA statement [29].

We carried out a systematic search on four major citation databases which were Medline, Cinahl, Cochrane Central Register of Controlled Trials and PubMed from 2014 to 2019 to include all trials published after the publication of previous systematic review in 2015 [26] which had covered studies up to 2013 [30]. The studies included were RCTs and have fulfilled the following inclusion criteria: participants must be adult; participants in intervention group practising qigong; comparators were control or exercise; and the outcome was the mean difference of systolic and diastolic blood pressure. The search strategy is shown in Additional file 1: Appendix 1.

### Intervention and control group definitions

Intervention groups included participants who were practicing qigong (or qi gong) while control groups included participants who were not subjected to any additional interventions or were only undertaking conventional exercise. The six types of qigong included as intervention in this study are the conventional qigong, Guolin Qigong, Shuxinpingxue gong, Dongeui Qigong, Ba Duan Jin Qigong and Mawangdui Daoyinshu Qigong. Guolin Qigong is also known as the Walking Qigong characterized by slow walking exercise in combination with coordinated arm movements and twisting of the waist [25] while Shuxinpingxue gong is a subtype of qigong that calms the heart by regulating the blood pressure [31]. Mawangdui Daoyinshu Qigong is another type of qigong that consists of a set of 12 coordinated movements [32] and Ba Duan Jin Qigong is also known as the Eight section Brocade incorporating eight types of movements [33]. We excluded trials if the interventions in the qigong or control group contained other non-conventional therapies such as acupuncture, traditional medication including herbs and also massages.

### Study selection

We searched for randomized controlled trials that reported the effects of qigong on both the systolic and diastolic blood pressure readings. The titles and abstracts were screened for potentially relevant RCT which must be published in the English language or have a full English translation with full text availability. The full texts were then retrieved for further data extraction.

### Data extraction

Two investigators (MNR and NAS) independently reviewed and performed data extraction. We extracted essential data such as: first author, publication year, location of study, the sample size of two groups, mean age of participants, duration of qigong, types of qigong, main findings and adverse effects. Per protocol principle was used for all outcomes, in which we used the number of participants who had completed treatment in each of the trial arms and performed the analyses based on these numbers [34]. Therefore, participants who were lost to follow-up were excluded from the final analysis regardless of the initial randomization process.

### Data synthesis

We used mean ± standard deviation (SD) to report our data. If the mean difference and SD was not available, calculation of the mean was performed by subtracting the mean of baseline measurement from the corresponding mean of post interventional measurement; while SD was imputed from the end point measurements. If the mean difference was reported in the trials, without the SD, the latter was imputed either from the end point measurements or calculated using the formula “SQRT (sample size)*(upper confidence interval-lower confidence interval)/(T.INV.2T(0.05, $D$2-1)*2)” as proposed by [35], in which this function was used to calculate the estimated SD with the Excel® software.

### Risk of bias assessment

Two reviewers (YSL, SKV) used the RoB 2: A revised Cochrane risk-of-bias tool for randomized trials to independently assess the risk of bias within each study [36]. These two reviewers assessed all the sources of bias. Any disagreements between these both reviewers were resolved by having a consensus with the other members of the review team (SMC, JXN, JLW, FKH and NKD).

### Statistical analysis

We used Review Manager (RevMan®) version 5.3 and trial sequential analysis for statistical analysis. Meta-analyses were performed using a random effects model to estimate the pool effect in term of weighted mean difference (WMD) and 95% confidence intervals (CI). Heterogeneity between trials was assessed by using the I^2^ statistic [34]. We assessed the publication bias using funnel plot and small-study effects using Egger’s regression test. The per-protocol principle was used for all analyses. A two-sided *p*-value < 0.05 was considered to be the cut off point for statistical significance.

Type-I errors may occur in meta-analyses which may lead to an increased risk of random errors. This happens when a cumulative meta-analysis is updated with the latest RCTs whereby smaller numbers of patients and few RCTs are included as part of the analysis [28, 37]. Therefore, we conducted trial sequential analysis (TSA) using TSA software package (available at http://www.ctu.dk/tsa/ [38]. It gives an adjusted threshold for statistical significance together with a combination of information on the cumulated sample size estimation for the cumulative meta-analysis.

### Grade

We used the Grading of Recommendations Assessment, Development and Evaluation (GRADE) to evaluate the quality of evidence of outcomes from the following 5 aspects: limitations, inconsistency, indirectness, imprecision, and publication bias. The quality of evidence would be graded as “high,” “moderate,” “low,” or “very low.”

## Results

### Description of included trial

**Fig. 1:**
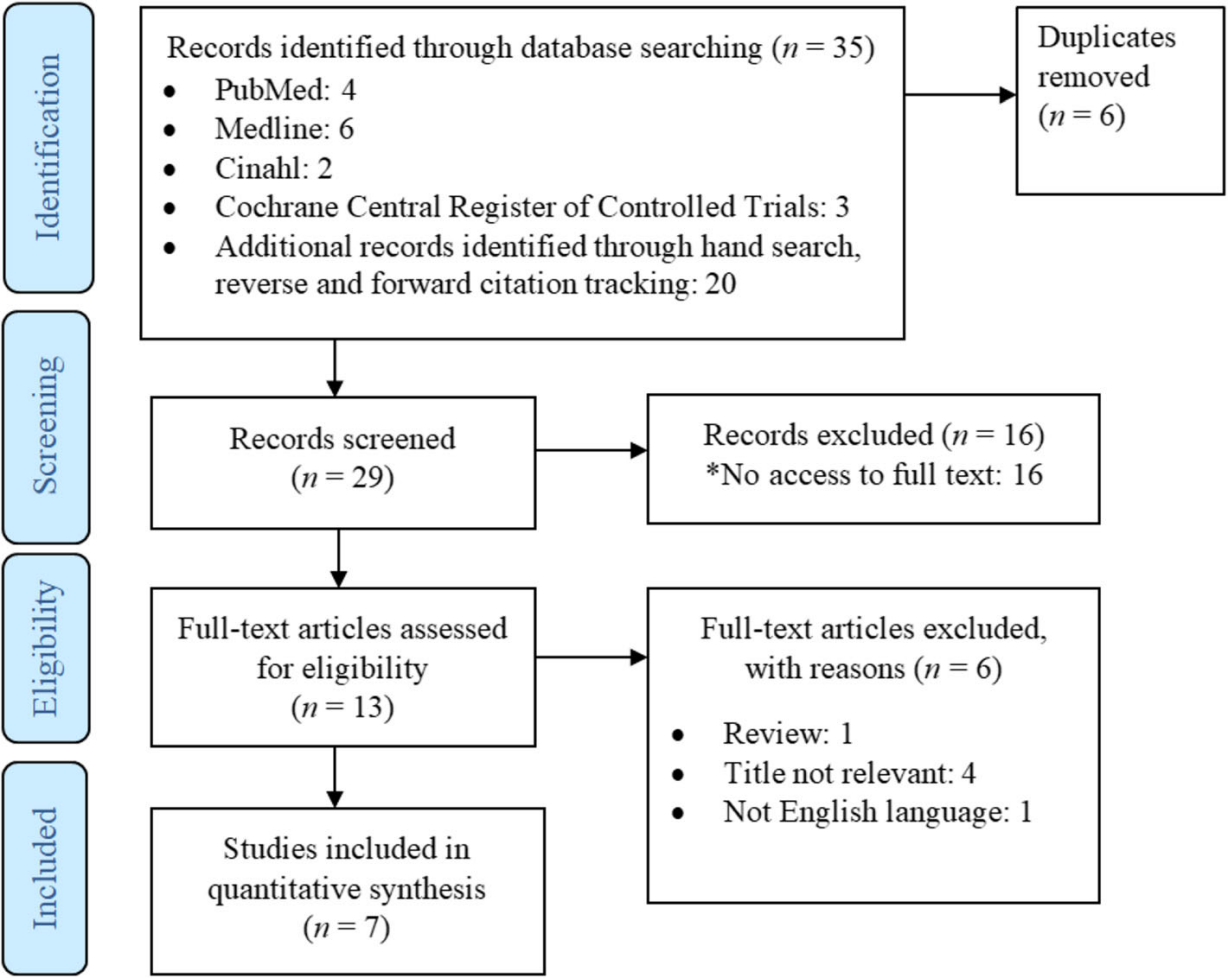
PRISMA flow diagram of study selection and identification

PRISMA flow diagram for study selection is presented in Fig. 1. A total of seven trials were included into the analysis as shown in Table 1.

**Table 1:** Basic Characteristics of the Included Studies

Author	Year	Country	Population Characteristic	Mean Age	Intervention (T)	Control©	Intent-ion to treat	Per protocol (Qigong/ Control)	Trial Duration	Amount of practice	Duration of Qigong	Outcome
Lee et al	2003	Korea	Essential hypertension	T: 55.8 ± 6.3 C: 57.1 ± 7.6	Shuxin-pingxue gong	Wait-list control	65	58 (29/29)	8 weeks	Not mention-ed	30 min/d included the warming-up (5 minutes), the Qigong (20 min) and the cool-down exercise (5 min)	Qigong may stabilize the sympathetic nervous system, improve ventilatory functions and reduce BP positively in mildly hypertensive middle-aged patients
Lee et al	2004	Korea	Essential hypertension	T: 52.6 ± 5.1 C: 54.3 ± 5.5	Shuxin-pingxue gong	Wait-list control	46	36 (17/19)	8 weeks	Twice per week	30 min/d, including warm-up (5 minutes), the qigong exercise (20 min), and cool-down exercises (5 min)	Qigong has positive effects on reducing BP and enhancing perceptions of self-efficacy
Cheung et al	2005	Hong Kong	Essential hypertension	T: 57.2 ± 9.5 C: 51.2 ± 7.4	Guolin Qigong	Conventional exercise: 60 min in the morning and 15 min in the evening/d	91	76 (37/39)	16 weeks	Daily	60 min in the morning and 15 min in the evening/d	There is no significant difference in the effects of qigong and conventional exercise on BP in patients with mild hypertension
Ji-eun Park et al	2014	Korea	Pre-hypertension and mild hypertension.	52.0 (43.0, 61.0)	Qigong	No interventi-on	44	40 (19/21)	8 weeks	More than 2 times per week	30 min per session	Qigong presented statistically significant effects compared with no treatment in decreasing BP for prehypertension and mild essential hypertension
Chen D	2016	Beijing	Essential hypertension	66.3 ± 5.8	Mawangdui Daoyinshu Qigong (MDQ)	No interventi-on	60	60 (30/30)	6 months	Five times per week	warm-up (5 min), the Qigong exercise (34 min), and a cool-down (5 min	MDQ may have the potential to improve SBP, DBP, HDL-C, LDL-C, TPR, FG, serum NO, and plasma ET-1.
Xiao et al	2016	Beijing	Essential hypertension	65.6 ± 7.8	Ba Duan Jin Qigong	No interventi-on	48	48 (24/24)	6 months	Five times per week	5-min warm-up, 30 min of Qigong exercise, and a 5-min cool-down.	Ba Duan Jin Qigong exercise may have the potential to improve SBP, DBP, HDL-C, LDL-C, TC, triglycerides, fasting glucose, serum NO, and plasma ET-1.
Ji-Eun Park et al	2017	Korea	Pre-hypertension and mild hypertension.	54.52 ± 6.96	Dongeui Qigong	No interventi-on	74	52 (25/27)	12 weeks	More than five times a week	Qigong class lasted 50 min,consisted of a warm-up (15 min),main qigong treatment (25 min), and a cool-down (10 min)	Dongeui qigong treatment for 12 weeks is not significantly effective in prehypertension and mild hypertension compared with no treatment

Data are presented in mean ± standard deviation or (minimum value, maximum value), *T* Treatment group, *C* control group

### Participant characteristics

The mean age of the participants ranged from 52.0 years to 65.6 years. Two trials [39, 40] were conducted among both prehypertension and mild hypertension groups while the rest [32, 41–44] were conducted on patients with essential hypertension. Two studies were conducted in Beijing [32, 44], four in Korea [39–42] and one in Hong Kong [43].

### Intervention characteristics

Seven studies exclusively discuss qigong exercise which include their own curated set of warm up, cool down exercises and a variety of qigong exercises. In total, 181 patients practised qigong while 181 patients did not receive any additional intervention [32, 39–42, 44]. The total duration of qigong ranged from 30 to 60 min which also showed varying duration of the warm up and cool down sessions that were implemented. The amount of practice of qigong is reflected by the overall treatment duration ranging between 8 weeks to 24 weeks and also the frequency of qigong practised. Six of the seven studies report varying frequency of qigong practised (on a weekly basis) ranging from twice per week up to daily [32, 39, 40, 42–44]. One study had no mention of practice frequency [41].

### Risk of bias assessment

**Fig. 2:**
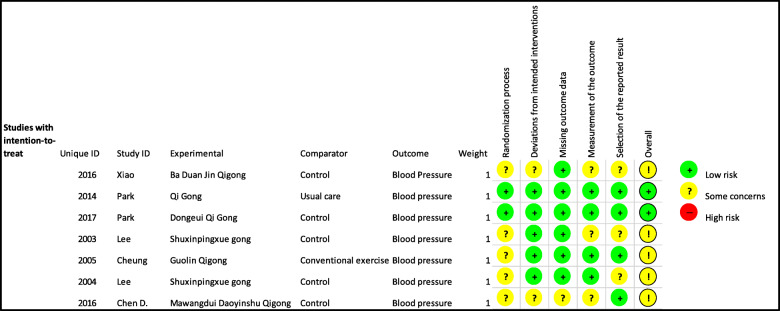
Risk of Bias according to RoB 2: A revised Cochrane risk-of-bias tool for randomized trials

We assessed the risk of bias of the included studies based on the process that was elaborated in the methods section using five pre-determined domains which included the following domains: bias arising from the randomization process, bias due to deviations from intended interventions, bias due to missing outcome data, bias in measurement of the outcome and bias in the selection of the reported results. Out of the seven trials included, two trials had a low risk of bias [39, 40]. The other five were all classified as having some concern or uncertain risk of bias [32, 41–44]. These five trials were classified as having some concern of bias due to a lack of information regarding the process of randomization and allocation concealment. A summary of the risk of bias assessment is as shown in Fig. 2.

### Weighted mean difference of blood pressure

#### Systolic blood pressure (SBP)

**Fig. 3:**
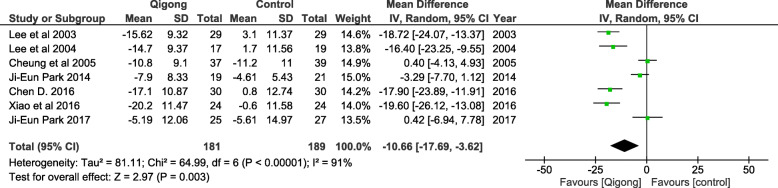
The effect of Qigong on reduction of systolic blood pressure

Meta-analysis using the seven eligible RCTs (*n* = 370) revealed that the practice of qigong had a significant effect in the lowering of SBP as compared to control [WMD, − 10.66 mmHg (95% confidence interval (CI) = − 17.69,-3.62, *p* < 0.001), with a significant heterogeneity of I^2^ = 91% (Fig. 3).

#### Diastolic blood pressure (DBP)

**Fig. 4:**
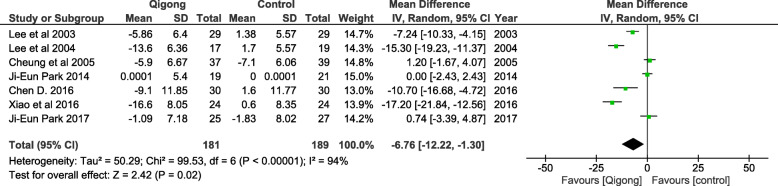
The effect of Qigong on reduction of diastolic blood pressure

Meta-analysis done on the effect of qigong on diastolic blood pressure reduction indicates a statistically significant reduction when compared to the control group (WMD = − 6.76 mmHg, 95% CI = − 12.22, − 1.30, *p* < 0.001). In this analysis, there was also significant heterogeneity observed with a I^2^ = 94% as shown in Fig. 4.

Any evidence of publication bias was observed by using the Funnel plot asymmetry test for the assessment of SBP (Additional file 1: appendix 2) and DBP (Additional file 1: appendix 3) using the WMD. Due to the limitation in the form of the number of included trials in the meta-analysis (less than 10 trials), funnel plots could not be applied for both SBP and DBPs to detect any potential publication bias. Thus, we conducted Egger’s regression test which demonstrated no evidence of small-study effects (Additional file 1: Appendix 4 and 5). The Egger’s regression test indicated no significant publication bias for both SBP (*p* = 0.269) and DBP (*p* = 0.117), although the number of included studies was small.

### Trial sequential analysis (TSA) for effect of qigong on blood pressure

#### Systolic blood pressure

**Fig. 5:**
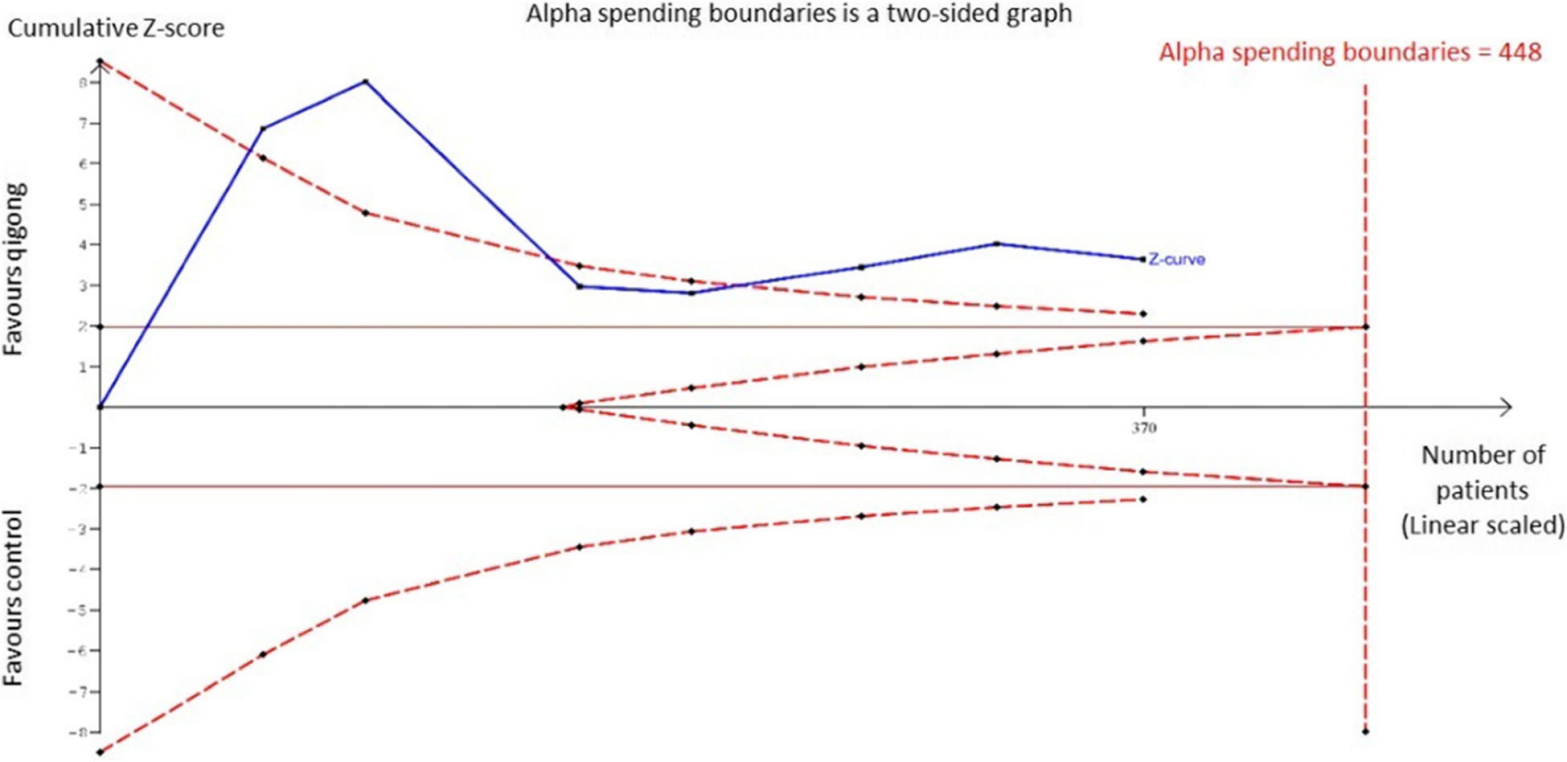
Trial Sequential analysis on the effect of Qigong on systolic blood pressure reduction

TSA on the effect of qigong on SBP readings is shown in Fig. 5. The required heterogeneity-adjusted information size to demonstrate or reject the weighted mean reduction (as per Fig. 3) of SBP is 516 patients (based on the meta-analysis of trials with a low risk of bias with an alpha (type-1 error) value of 5% and a power of 80%). The cumulative Z-curve (blue curve) crossed the conventional boundary (Z-statistic above 1.96) and demonstrated firm evidence that Qigong has statistically significant lowering effect on SBP as indicated in our meta-analysis. However, the number of patients included in our meta-analysis did not cross the alpha spending boundaries showing that it did not meet the required information size which was 448 patients. This indicates that the positive lowering effect of qigong on systolic blood pressure remains inconclusive based on only 370 participants.

#### Diastolic blood pressure

**Fig. 6:**
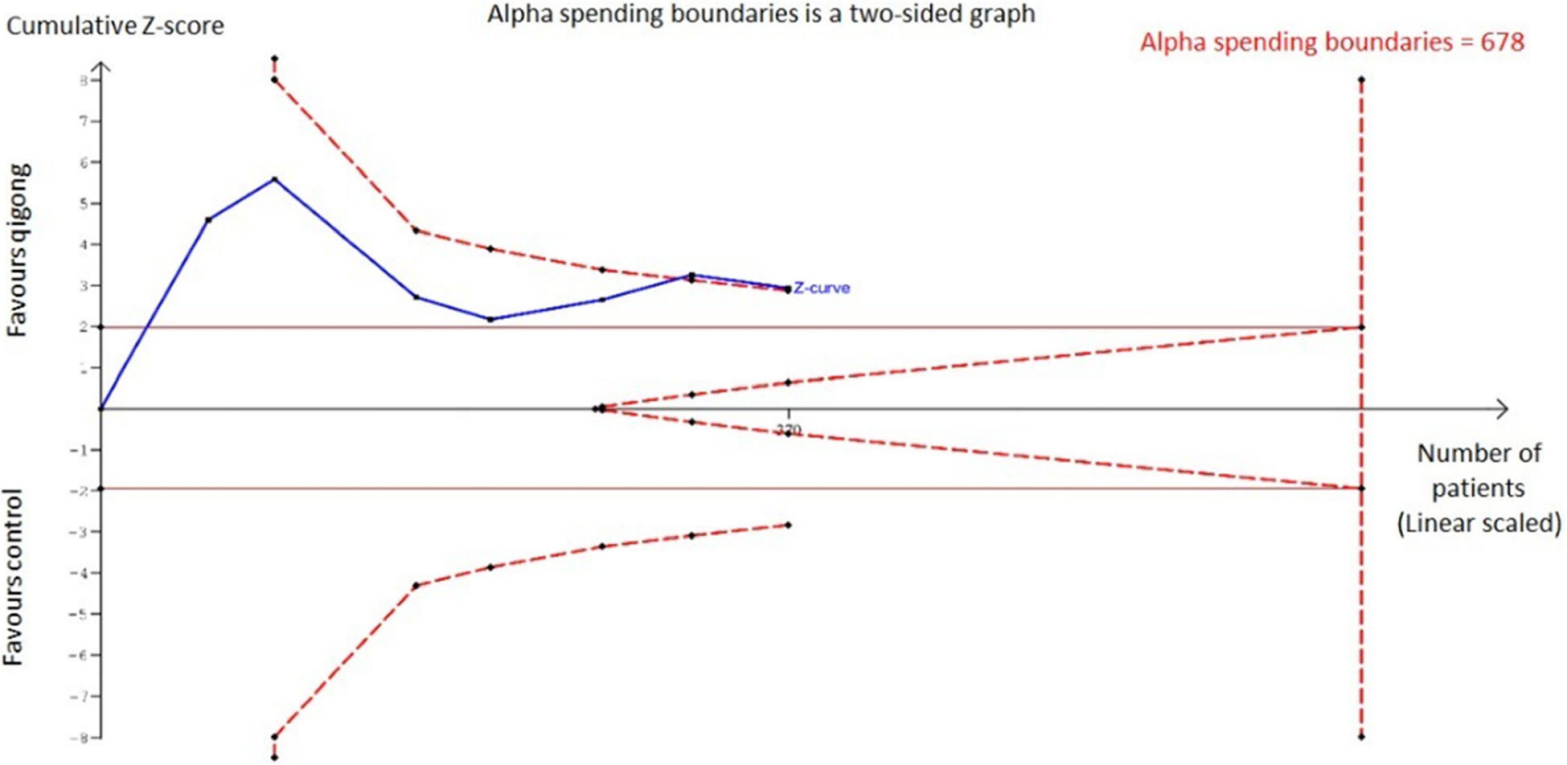
Trial Sequential analysis on the effect of Qigong on diastolic blood pressure reduction

From the TSA, the required heterogeneity-adjusted information size to demonstrate or reject the weighted mean reduction (as per Fig. 4) of DBP is 516 patients (based on the meta-analysis of trials with a low risk of bias with an alpha (type-1 error) value of 5% and a power of 80%). The cumulative Z-curve (as shown in Fig. 5) has crossed the conventional boundary (Z-statistic above 1.96) with all the trials included demonstrating a firm evidence that the diastolic blood pressure reduction effect of qigong is statistically significant. However, the number of patients from all the trials included into this analysis does not fulfill the required information size of 678 participants as shown by the cumulative Z-curve (blue line) not crossing the alpha spending boundary. This indicates that with a sample of 370 participants, the effect of qigong on diastolic blood pressure reduction remains inconclusive. This can be seen in Fig. 6 below.

### Quality of evidence

Using the GRADE summary of evidence, the quality of evidence for the SBP of qigong vs control and DBP of qigong VS control is shown in Additional file 1: Appendix 6.

## Discussion

This is an updated systematic review of effects of qigong on systolic and diastolic blood pressure lowering since the last systematic review that was last published in 2015 [26] and 2007 [27]. The first systematic review in 2007 by Lee et al. included 12 trials with 1218 participants while the subsequent systematic review which was conducted by Xiong et al. in 2015 has included 20 trials with 2349 participants. To our knowledge, we are the first to perform the trial sequential analysis of the comparative effectiveness of qigong exercise on SBP and DBP blood pressure reduction. The currently available best evidence for the prevention of hypertension based on the results of RCTs and meta-analysis in this study reflected that qigong is superior as compared to when no intervention is applied. We found a total of seven eligible studies and identified several novel findings that we can use to update the previous review and provide more conclusive evidence on the beneficial effect of qigong on controlling blood pressure. Despite having different methodologies, most studies were consistent in the conclusion that the practice of qigong does reduce the systolic and diastolic blood pressure as compared to control groups.

In comparison with the previous meta-analysis [45], which reported on trials up to 2013 [30], our study added another four trials that were published from 2014 to 2017 in order to update the systematic evidence of positive effects of qigong practice in the ever growing world of medicine. In addition, we performed a trial sequential analysis, which was able to provide confirmation and firm evidence of the effect of qigong on blood pressure reduction. The two previous systematic reviews were unable to draw conclusions to the effectiveness of qigong. From our TSA analysis, we are able to provide firm evidence of the effect of qigong as being able to reduce SBP and DBP blood pressures significantly. However, this analysis also demonstrates that there were difficulties in drawing a firm conclusion considering the shortfall in meeting the required information size.

Moreover, with the previous meta-analyses [26, 27], most of their trials were presented as having a high risk of bias. This could be due to the fact that there could be inclusion of unpublished trials from their searches through trial registries that could have given rise to a high publication bias. The studies included in our updated review show two out of the seven studies as having low risks. These studies with a clear methodology have all been published and are available from established search engines.

Our results indicate an interesting aspect of qigong practice. According to the analysis, qigong is capable of reducing systolic and diastolic blood pressure as a stand-alone alternative management tool and adjunctive therapy as evidenced by the findings from the TSA. These findings could be explained by its underlying mechanism which are adjunctive and not independent of one another, incorporating all of its three elements [45, 46]. This finding is in line with previous related research results that have been reported on the effects of qigong exercise in reducing blood pressure in hypertensive patients [45, 47].

In terms of mechanism within contemporary frameworks, qigong is capable in enhancing interoception, regulating the cognitive control network, and modulating emotion processing to boost well-being [46]. It can be considered as a mindful exercise which leads to changes in central neurotransmitter systems, neuroendocrine modulation and reduction in stress-related hormones and regulation of neurotrophic factors and central neuroplasticity [48]. As reflected by the cardiovascular fitness construct, qigong mechanisms may involve regulation of the autonomic nervous system with optimization of SNS activity, increases in PNS activity and enhanced sympatho-vagal balance through its enhanced slow-breathing effect which then decreases the heart rate [48, 49]. This may then lead to the down-regulation of the sympathetic and up-regulation of the parasympathetic nervous system that can have a positive lowering impact on high blood pressure [50].

Besides its main domain of benefit with hypertension, qigong also benefits in the management of other health issues. Qigong is also known to have potentials in other health modalities. Qigong exercise is an efficacious and acceptable treatment for sleep disturbance, fatigue, anxiety, and depressive symptoms in people with chronic fatigue [51]. Moreover, it has also been recommended to assist adults with a variety of intractable chronic musculoskeletal painful conditions to achieve a better quality of life and improved pain status with the potential of wide-reaching physical and psychological benefits for ameliorating pain. Qigong, which encompasses physical and cognitive features, allows it to be recommended as a salient non-invasive non-pharmacological treatment for chronic pain conditions [52]. Qigong is also recommended as it has moderate-to-strong effects on pain, quality of life, sleep quality and moderate effects on fatigue in cases of fibromyalgia syndrome [53]. Additionally, qigong is known in the field of cancer treatment whereby it has positive effects on the cancer-specific quality of life, fatigue, immune function and cortisol level of cancer patients [54].

The adverse effect of qigong is almost none as evidenced by this updated review and previous reviews. The results of this study showed that qigong offers an alternative in the management of hypertension and can help in improving patient’s quality of life especially in the elderly population group who occasionally have a tendency to prefer non-pharmacological management due to polypharmacy in this age group. The use of TSA confirmed the evidence that qigong does indeed help in reducing systolic and diastolic blood pressure with the addition of four more trials as compared to the previous meta-analysis [27, 45]. However, it is suggested that the practice of qigong should be tailored according to patient’s condition and fitness, preferences, and cultural needs as well as cost implications accrued for both the patient and the government in organizing professional teaching courses conducted by experts. TSA has further confirmed that qigong does indeed reduce systolic and diastolic blood pressure and therefore should be considered for specific targeted groups. Conclusively, we would like to recommend qigong as an adjunct or compliment to medical treatment for blood pressure control or as undertaken as part of a medically less supervised program where efficiencies in delivery will occur due to group practice.

### Limitations

The heterogeneity of this study’s RCTs is an expected problem that was difficult to address. Considering all seven trials, there were differences in the type of qigong practised, in the duration of the trial as well, in the frequency of qigong practice as well as the level of expertise in teaching the exercise. There are gaping differences in the study populations, including the location of study and patient-related variables such as other concurrent medical problems and the severity of the hypertension. For example, only two studies [32, 44] out of the seven trials were from China, said to be the origin of qigong practice, where there are high standards of facilities and access to treatment considering China is said to be the origin for the practice of qigong.

Another limitation is the inability to expand our sample size as evidenced from the TSA where the required sample size could not be met. This can be attributed to the fact that a fair amount of potentially eligible trials was in the Chinese language from websites that were either non-accessible or not available for full text viewing. Besides, the possibility of some of the trials being unpublished prompted the exclusion of these Chinese trials in this study as it can contribute to further publication bias.

The risk of bias of most of the trials was due to concerns arising from the inadequate details regarding randomization and allocation concealment. However, poor allocation concealment does not affect directly on the results reported by qigong trials as general and also those included in this study as concealment is a complex component to tackle in interventions that deal with practices as opposed to pharmacological treatments. As such, the results should still be interpreted with caution. Rigorously designed and high-quality trials are highly warranted to confirm the results of this study. There is lack of firm evidence for a beneficial effect and an insufficient information size to accept the anticipated intervention effect.

## Conclusions

The overall results of this systematic review, powered with TSA, have shown that qigong does reduce systolic and diastolic blood pressure and it can be a viable complementary therapy in the management of hypertension. Considering the sample size of the studies included in this systematic review, additional large well-designed randomized trials with a low risk of bias are warranted to promote qigong as an effective strategy to augment the blood pressure control of hypertensive patients.

## Supplementary Information

**Additional file 1: Appendix 1** Search Analysis. **Appendix 2** Assessment of Systolic Blood Pressure Weighted Mean Difference (The Funnel Plot Asymmetry Test). **Appendix 3** Assessment of Diastolic Blood Pressure Weighted Mean Difference (The Funnel Plot Asymmetry Test). **Appendix 4**: Assessment of small-study effects by using Egger’s regression test for the effect of Qigong on reduction of systolic blood pressure. **Appendix 5**: Assessment of small-study effects by using Egger’s regression test for the effect of Qigong on reduction of diastolic blood pressure. **Appendix 6**: Results of GRADE.

## Data Availability

All data generated or analysed during this study are included in this published article [and its supplementary information files].

## Abbreviations

RCT: Randomized controlled trials
SBP: Systolic blood pressure
DBP: Diastolic blood pressure
TSA: Trial sequential analysis
WMD: Weighted mean difference
