# Supplementary material for: Effects of qigong on systolic and diastolic blood pressure lowering: a systematic review with meta-analysis and trial sequential analysis
Source: BMC Complement Med Ther. 2021 Jan 6;21:8. doi: 10.1186/s12906-020-03172-3 (PMC7789757; doi:10.1186/s12906-020-03172-3)
Supplement: Supplementary file 1 — Additional file 1: Appendix 1 Search Analysis. Appendix 2 Assessment of Systolic Blood Pressure Weighted Mean Difference (The Funnel Plot Asymmetry Test). Appendix 3 Assessment of Diastolic Blood Pressure Weighted Mean Difference (The Funnel Plot Asymmetry Test). Appendix 4: Assessment of small-study effects by using Egger’s regression test for the effect of Qigong on reduction of systolic blood pressure. Appendix 5: Assessment of small-study effects by using Egger’s regression test for the effect of Qigong on reduction of diastolic blood pressure. Appendix 6: Results of GRADE. [file 12906_2020_3172_MOESM1_ESM.docx]

**Appendices**

Appendix 1 Search Analysis

| No | Search | Pubmed | Cinahl | Medline | Cochrane |
| --- | --- | --- | --- | --- | --- |
| 1 | exp QIGONG/ | 689 | 135 | 211 | 363 |
| 2 | exp QI GONG/ | 100 | 2 | 28 | 25 |
| 3 | Exp HYPERTENSION/ | 381985 | 6474 | 60081 | 40838 |
| 4 | exp BLOOD PRESSURE/ | 298634 | 3484 | 59229 | 73830 |
| 5 | exp Randomized Controlled Trial as Topic/ | 68555 | 13498 | 46013 | 126425 |
| 6 | Exp Clinical Trial/ | 1172245 | 6841 | 107180 | 88493 |
| 7 | random$.ab. | 265789 | 633 | 60843 | 1441 |
| 8 | 3 or 4 | 581732 | 9616 | 101970 | 34837 |
| 9 | 1 or 2 | 784 | 137 | 239 | 368 |
| 10 | 5 or 6 or 7 | 1424332 | 1930 | 176514 | 331898 |
| 11 | 8 and 9 and 10 | 34 | 14 | 12 | 15 |
| 12 | limit 11 to (humans and yr="2014 - 2019") | 4 | 2 | 6 | 3 |

Appendix 2 Assessment of Systolic Blood Pressure Weighted Mean Difference (The Funnel Plot Asymmetry Test)


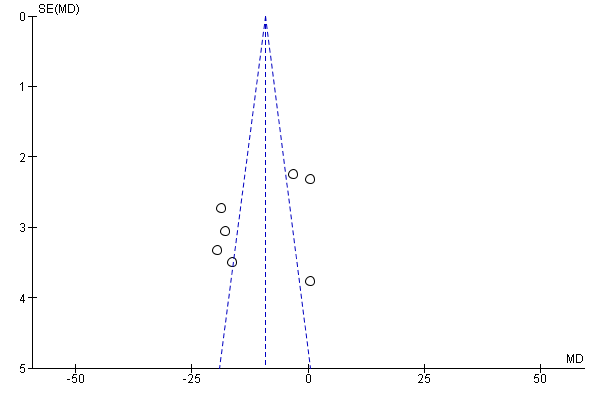


Appendix 3 Assessment of Diastolic Blood Pressure Weighted Mean Difference (The Funnel Plot Asymmetry Test)


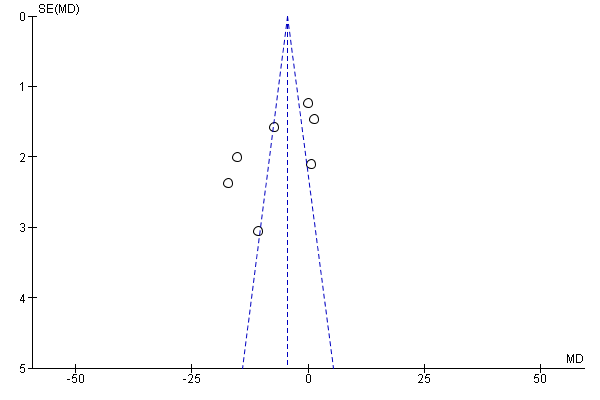


Inference: Presence of publication bias due to evidence of asymmetry in the funnel plot. Due to the limited number of studies (<10 studies) included, these results should be interpreted with caution.

Appendix 4: Assessment of small-study effects by using Egger’s regression test for the effect of Qigong on reduction of systolic blood pressure.

Appendix 5: Assessment of small-study effects by using Egger’s regression test for the effect of Qigong on reduction of diastolic blood pressure

Appendix 6: Results of GRADE

| Results of GRADE | | | | | | | | | | | | |
| --- | --- | --- | --- | --- | --- | --- | --- | --- | --- | --- | --- | --- |
| **Certainty assessment** | | | | | | | **№ of patients** | | **Effect** | | **Certainty** | **Importance** |
| **№ of studies** | **Study design** | **Risk of bias** | **Inconsistency** | **Indirectness** | **Imprecision** | **Other considerations** | **Qigong** | **Control** | **Relative (95% CI)** | **Absolute (95% CI)** |  |  |
| **Systolic (assessed with: mmHg)** | | | | | | | | | | | | |
| 7 | randomised trials | not serious | serious | not serious | not serious | publication bias strongly suspected | 181 | 189 | - | MD **10.66 mmHg lower** (17.69 lower to 3.62 lower) | ⨁⨁◯◯ LOW | IMPORTANT |
| **Diastolic (assessed with: mmHg)** | | | | | | | | | | | | |
| 7 | randomised trials | not serious | serious | not serious | not serious | publication bias strongly suspected | 181 | 189 | - | MD **6.76 mmHg lower** (12.22 lower to 1.3 lower) | ⨁⨁◯◯ LOW | IMPORTANT |

**CI:** Confidence interval; **MD:** Mean difference
